# A saúde da população negra e as políticas no século XX: é nas encruzilhadas que encontramos resistências, emancipações e mortes

**DOI:** 10.1590/0102-311XPT080224

**Published:** 2025-04-11

**Authors:** Ivison Luan Ferreira Araújo, Luiz Paulo Ribeiro

**Affiliations:** 1 Universidade Federal de Minas Gerais, Belo Horizonte, Brasil.

**Keywords:** Negro, Políticas, Racismo, Direito ao Cuidado de Saúde, Blacks, Policy, Racism, Right to Health Care, Negro, Políticas, Racismo, Derecho a la Atención Sanitaria

## Abstract

O movimento negro, por meio da luta antirracista, educa e reeduca a população brasileira, exercendo influências sobre as condições de saúde a partir de ações que visam o bem-estar da população negra. O objetivo deste ensaio é identificar a relação da saúde da população negra com a política de morte e emancipatória a partir das perspectivas históricas, considerando o racismo no Brasil, o Estado e a resistência da população negra para a garantia do direito à saúde, do século XX até o processo de implementação da Política Nacional de Saúde Integral da População Negra (PNSIPN), em 2006. Este estudo parte de uma metodologia de ensaio teórico, possibilitando reflexões profundas sobre as perspectivas históricas relacionadas à saúde da população negra. Sendo assim, observa-se que existe uma continuação de mecanismos a partir da política de morte do Estado, prejudicando o acesso à saúde da população negra, devido aos racismos e suas ferramentas. Por outro lado, destaca-se que a população negra tem construído, durante anos, saberes e políticas emancipatórias a partir de intervenções sociais, culturais e políticas de forma intencional e direcionada, como a PNSIPN e o *Estatuto da Igualdade Racial*.

## Abstract (en)

The black movement uses the fight against racism to educate and reeducate Brazilian society, influencing health conditions with actions aimed at the well-being of the black population. This essay aims to identify the relationship between black population’s health and death and emancipation policies from a historical perspective, considering racism in Brazil, the role of the state, and black resistance in guaranteeing health rights, from the twentieth century to the implementation of the Brazilian National Comprehensive Health Policy for the Black Population (PNSIPN, acronym in Portuguese) in 2006. This study adopts a theoretical essay methodology, enabling in-depth reflections on historical perspectives related to black people’s health. The findings suggest a continuation of mechanisms based on the state’s death policies that hinder black communities’ access to health due to racism and its tools. At the same time, the black population has actively built emancipatory knowledge and policies over the years with intentional social, cultural and political interventions, such as the PNSIPN and the *Brazilian Racial Equality Statute*.

## Abstract (es)

El movimiento negro mediante la lucha antirracista educa y reeduca a la población brasileña, lo cual ejerce influencias sobre las condiciones de salud basadas en acciones dirigidas al bienestar de la población negra. El objetivo de este ensayo es identificar la relación entre la salud de la población negra y la política de muerte y emancipación desde perspectivas históricas, teniendo en cuenta el racismo en Brasil, el Estado y la resistencia de la población negra para garantizar el derecho a la salud desde el siglo XX hasta el proceso de implementación de la Política Nacional de Salud Integral de la Población Negra (PNSIPN) en 2006. Este estudio parte de una metodología de ensayo teórico para fomentar reflexiones profundas sobre las perspectivas históricas relacionadas con la salud de la población negra. Para ello, se observa que existe una continuación de mecanismos basados en la política de muerte del Estado, lo que perjudica el acceso a la salud de la población negra, debido al racismo y sus mecanismos. Por otro lado, cabe destacar que, durante años, la población negra viene construyendo conocimientos y políticas emancipadoras a partir de intervenciones sociales, culturales y políticas de manera intencional y dirigida, como la PNSIPN y el *Estatuto de Igualdad Racial*.

## Introdução

Os movimentos negros, por meio da luta antirracista, demonstram, problematizam, educam e reeducam a população brasileira a partir da interpretação da raça e suas estruturas, passando a ocupar espaços nas análises sociológicas e na construção de políticas públicas desde sua criação ^1^. Assim, exercem influência sobre as condições de saúde a partir de ações que visam o bem-estar da população negra ^2^, com articulações que perpassam, por exemplo, a luta do movimento de mulheres negras e a Marcha Zumbi dos Palmares contra o Racismo, pela Cidadania e a Vida, realizada em 1995. Esses movimentos obtiveram resultados importantes em relação ao direito sexual e reprodutivo das mulheres e à valorização da população negra, a partir de discussões sobre o racismo e seus efeitos no que diz respeito à falta de acesso ao Sistema Único de Saúde (SUS) ^3^ ^,^ ^4^ ^,^ ^5^.

Assim, o movimento negro, como um todo, é um ator coletivo e político que emancipa a sociedade, seja pelo fato de todos e todas serem herdeiros(as) da sabedoria ancestral individual e coletiva ou pelo conhecimento nascido na luta ^1^. Ou seja, os negros e negras do Brasil têm saberes e conhecimentos emancipatórios produzidos ao longo do tempo que, abraçados pelos movimentos negros, são articulados e sistematizados para a garantia de direitos.

Depois de intensas lutas coletivas dos movimentos negros, surgiu o processo de construção do Grupo de Trabalho Intersetorial para a Valorização da População Negra; já em âmbito internacional, ocorreu a III Conferência Mundial contra o Racismo, Discriminação Racial, Xenofobia e Intolerância Correlata, em 2001, na qual foi reconhecida a ausência do Ministério da Saúde brasileiro no enfrentamento das questões raciais, o que reforçou a necessidade de estudos sobre a saúde da população negra ^6^. Esses dois grandes processos de luta serviram como experiências importantes para a construção da Política Nacional de Saúde Integral da População Negra (PNSIPN), aprovada pelo Conselho Nacional de Saúde (CNS) no ano 2006 e efetivada pela *Portaria nº 992/2009* ^7^, cujo objetivo é promover a saúde integral da população negra, priorizando a redução das desigualdades étnico-raciais, o combate ao racismo e à discriminação nas instituições e serviços do SUS ^8^.

A PNSIPN partiu de articulações e processos emancipatórios dos movimentos e da população negra, como mencionados anteriormente, garantindo um avanço no cuidado. Entretanto, apesar de se estabelecer a partir de um processo político emancipatório, essa política não tem sido efetivada em todos os municípios brasileiros, por causa do processo de racismo institucional e estrutural; ou seja, mesmo com o avanço e a importância dessa política, ainda existem grandes dificuldades. A população ainda sofre com a disparidade e o tratamento desigual no SUS, exercendo, por exemplo, diferenças na prevalência de algumas doenças e taxas de mortalidade ^9^.

O racismo é estruturador das relações sociais, permeando um conjunto de práticas institucionais, históricas, culturais e interpessoais ^10^, não sendo diferente quando consideramos a saúde da população negra no Brasil, visto que o racismo é um dos determinantes sociais de processos de adoecimento e morte ^6^, indo ao encontro de uma política de Estado que mata, desumaniza e domina os corpos negros ^11^, além de regulá-los a partir do corpo escravo, estereotipado, objetificado ou mesmo cooptado pelo capitalismo industrializante. Porém, “*as negras e negros em movimento transformam aquilo que é produzido como não existência em presença, na sua ação política*” ^1^ (p. 15), emancipando seu corpo a partir da construção da identidade, do ser político e da estética do próprio povo negro.

Dessa forma, consideramos que tal discussão em relação ao racismo no SUS é fundamental para a construção de novas possibilidades e práticas que possam diminuir a estrutura do racismo e as desigualdades. Nesse sentido, este ensaio tem o objetivo de identificar a relação da saúde da população negra no século XX com a política de morte e emancipatória, sob uma perspectiva histórica identificada a partir do encadeamento de processos do passado, considerando o racismo no Brasil, o Estado e a resistência da população negra para a garantia do direito à saúde, do século XX até o processo de implementação da PNSIPN no Brasil, em 2006.

## A saúde da população negra no século XX

No século XX, a elite engajara-se numa verdadeira luta para apagar as marcas de uma herança de atraso e tradição, substituindo seus ideais por padrões europeus de civilização, com vistas a um futuro de ordem e progresso. Num cenário impregnado por higienistas e eugenistas, a grande reprovação era do trabalho das amas pretas, que sustentavam as instituições que acolhiam crianças órfãs ou abandonadas e realizavam suas atividades nas casas de famílias ricas, ou seja, a higienização da maternidade e da infância poderia representar a higienização da sociedade brasileira, materializando, dessa forma, a transformação social e política do país ^12^.

No início do século XX, a eugenia era uma ferramenta de controle populacional para a garantia da seleção social, representando a superioridade racial e o orgulho da identidade nacional, promovendo intervenções na vida social, no corpo e na reprodução humana, como a segregação racial, a eutanásia e o racismo genético, com o apoio de médicos, intelectuais, autoridades públicas, entre outros. A eugenia no Brasil foi incorporada em diferentes projetos científicos e políticos de seleção social e reforma nacional, num processo que culminou em sua institucionalização, com medidas como a criação de barreiras legais que impediam imigrantes indesejáveis (negros, asiáticos, árabes) ou a esterilização em massa das mulheres negras e de pessoas com transtornos mentais. Estes dois últimos pontos não foram definidos por leis, mas se estabeleceram no cotidiano das discussões e das práticas dos eugenistas ^12^.

Na saúde mental, os princípios eugênicos tiveram continuidade até os anos 1970, além do processo de medicalização, configurando-se como base para o surgimento dos hospícios no Brasil e sendo fundamentais para o desenvolvimento do capitalismo, tendo em vista a docilização dos corpos e o impacto de ideologias dominantes sobre grupos dominados, atrelados aos aparelhos repressivos do Estado ^13^. Já entre os anos 1947 e 1951, o Hospital Psiquiátrico do Juquery, no Estado de São Paulo, realizava intervenções cirúrgicas no cérebro de mulheres, somando 95% das cirurgias e atendendo, majoritariamente, às mulheres negras, indo ao encontro da teoria da eugenia e dos princípios racialistas da época ^14^.

Dentro desses processos, os movimentos sociais e políticos passaram a demandar a expansão da cobertura efetiva dos serviços, cobrando novas posturas ético-políticas para a garantia da universalidade e da integralidade no cuidado médico, além da reivindicação de inclusão de novas temáticas na graduação, como o acesso aos serviços de saúde, a pobreza, as relações médico e paciente. Sendo assim, os movimentos de promoção da saúde ligados a valores da democracia, cidadania e multiculturalismo ameaçaram o antigo padrão da formação médica, com o intuito de diluir a supremacia do poder relacionada às profissões, incluindo a medicina ^15^.

Nesse período, a imprensa negra teve o seu papel emancipatório, rompendo o imaginário racista, que atribuía inferioridade a partir do racismo científico, que permeava o final do século XIX e o início do XX. Em consonância com processos políticos emancipatórios, surgiu a Frente Negra Brasileira, em 1931, com a intenção de potencializar uma articulação nacional a partir da educação e do entretenimento, além de promover articulações por meio da vida social, política e cultural. Em 1936, tornou-se partido político e, em 1937, foi extinta, em obediência ao decreto de Getúlio Vargas, que criminalizava os partidos políticos. Já entre 1944 e 1968, foi instituído o Teatro Experimental Negro (TEN), com o objetivo de contestar a discriminação racial e resgatar a ancestralidade por meio do teatro e da educação ^1^.

Na década de 1950, surgiu a agência bilateral Serviço Especial de Saúde Pública (SESP), ampliando a cooperação técnica entre Brasil e Estados Unidos, combinando ações de medicina curativa e preventiva, saneamento, controle de doenças transmissíveis e educação sanitária. Havendo a incorporação de pesquisadores sociais, antropólogos e sociólogos, com o intuito de atender à zona rural, uma vez que a população estava desassistida pelo poder público, que estava preocupado apenas com o aumento da produtividade agrícola e o crescimento econômico ^16^.

No contexto de Guerra Fria, era estratégico que os Estados Unidos mantivessem relações com o Brasil. Dessa forma, a SESP atuou na extração da borracha na Amazônia e do minério de ferro no vale do Rio Doce, cujas populações sofriam com doenças endêmicas e falta de infraestrutura. Assim, era importante manter a classe trabalhadora saudável, para garantir o desenvolvimento, dando lugar, posteriormente, ao associativismo e ao cooperativismo, apesar da grande dificuldade em relação aos concentracionistas fundiários, que impediam um plano de reforma agrária para a melhoria da população rural em relação à saúde ^16^. Quanto ao Movimento Negro, de 1940 a 1960, atuou nas políticas educacionais, reivindicando o acesso da população negra à escola pública e a incorporação de discussões sobre raça. Na Ditadura Militar, porém, a questão racial perdeu lugar na educação nacional e retornou apenas em 1996 ^1^.

Já em 1956, consolidaram-se as disciplinas básicas, os componentes preventivos e psicossociais no estudo médico, além de terem sido criados departamentos de medicina preventiva em escolas médicas. Já no período de 1971, apareceram o internato médico e a residência médica como temas para as escolas médicas ^15^. Em 1975, foi instituído o Sistema Nacional de Saúde, a partir da *Lei nº 6.229*, consolidando a histórica separação entre a saúde pública e a medicina previdenciária. Já em 1978, na Conferência de Alma Ata, houve a aprovação da atenção primária como estratégia principal para garantir a saúde de todos. Nessa mesma década, surgiu uma nova concepção da medicina e foram difundidas novas ideias, relacionando problemas de saúde e determinantes sociais ^17^.

Em relação às questões étnico-raciais, em 1978, surgiu o Movimento Unificado Contra a Discriminação Étnico-Racial (MUCDR), que, posteriormente, em 1979, foi rebatizado de Movimento Negro Unificado (MNU), elegendo a educação e o trabalho como pautas para a luta contra o racismo. Nesse período da Ditadura Militar, houve uma expansão expressiva da medicina preventiva. Além disso, havia os Institutos de Aposentadorias e Pensões (IAPs), que se destinavam a garantir serviços de saúde para os trabalhadores com carteira assinada e, de outro lado, a saúde pública, que, por meio do Ministério da Saúde, destinava-se a pessoas pobres e residentes das zonas rurais, além das instituições filantrópicos de saúde auxiliadas pelo poder público ^17^.

Depois, em 1978, surgiu o Instituto Nacional de Previdência Social (INPS), com o intuito de ampliar a cobertura da medicina previdenciária, indo ao encontro das propostas liberais, pois o INPS contratava serviços privados para o atendimento de seus beneficiários. Já a saúde pública foi estrangulada financeiramente, tendo redução de porcentagem dentro do orçamento da União. Mesmo em 1960, quando tiveram início os “anos dos milagres”, a política econômica estava centrada na concentração de riqueza e na contenção de gastos públicos, prejudicando as condições de vida da população ^17^. Nesse sentido, as críticas e denúncias que recaíam sobre o INPS/Instituto Nacional de Assistência Médica da Previdência Social (INAMPS) e a luta pela redemocratização garantiram fôlego para o debate acerca da criação de um sistema nacional e público de saúde ^18^.

Já na década de 1980, a Reforma Sanitária trouxe novas discussões na medicina, como a expansão do ensino médico e dos hospitais universitários. Em 1990, foi instituída a Comissão Interinstitucional Nacional de Avaliação das Escolas Médicas, e, em 1996, foi definida a Lei de Diretrizes e Bases da Educação Nacional, tornando o ambiente institucional mais receptivo a transformações no ensino médico, apesar de que, até os dias atuais, ainda existem alguns dilemas entre a lógica do mercado privado, com a fragmentação por especialidades, e a consolidação de um direito social para o fortalecimento de um sistema público de saúde ^15^.

É importante ressaltar que o processo da Reforma Sanitária brasileira foi fruto das iniciativas de uma elite intelectual com ideário progressista, que assumiu posições estratégicas na estrutura do Estado. Nesse aspecto, a reforma contribuiu para a difusão do direito à saúde em prol da democracia e do controle social, apesar do capitalismo e das limitações impostas por outras instituições. Ou seja, nesse momento, o propósito era uma nova sociedade, que levasse em consideração políticas sociais e a formulação de um pacto de solidariedade entre o Estado e a população brasileira ^18^.

Nesse processo de pacto de solidariedade surgiu o SUS, trazendo em sua concepção princípios importantes, como a equidade, a universalidade e a integralidade. Favorecendo avanços na saúde a favor da população e dos direitos direcionados à saúde, um grande exemplo é a atenção básica em saúde, que apresenta resolutividade entre 80% e 90% em relação às necessidades de saúde ^19^. Nesse entrelaçar de períodos, em 1990, a América Latina era palco de movimentações sociais, políticas e econômicas, passando por uma reforma constitucional após o autoritarismo. Nesse momento, a raça ganhou uma centralidade no Brasil, ocorrendo a Marcha Zumbi dos Palmares contra o Racismo, pela Cidadania e a Vida, em 1995, levando para o Executivo federal um programa para a superação do racismo e da desigualdade étnico-racial, além de demandas relacionadas a ações afirmativas na educação superior, no mercado de trabalho e na saúde ^1^.

O Movimento Negro participou da III Conferência Mundial Contra o Racismo, a Discriminação Racial, a Xenofobia e Formas Correlatadas de Intolerância, em 2001, em Durban (África do Sul). Nesse momento, o Estado brasileiro reconheceu a existência de racismo institucional e se comprometeu a construir medidas para superá-lo. A partir desse processo, houve mudanças estruturais, como a criação da Secretaria de Políticas de Promoção da Igualdade Racial (SEPPIR), em 2003. Além da fundação, em 2000, da Associação Brasileiras de Pesquisadores Negros (ABPN), responsável por construir o Congresso Brasileiro de Pesquisadores Negros (COPENE). Em 2003, surgiu a lei que torna obrigatória o ensino de história e cultura afro-brasileira e africana, e, em 2008, foi incluída a temática indígena ^1^. Em 2006, o CNS aprovou a PNSIPN, efetivada pela *Portaria nº 992/2009* ^7^.

A PNSIPN tem como marco o reconhecimento do racismo, principalmente o institucional, além das desigualdades raciais. A política proporciona, até os dias atuais, o desenvolvimento de ações especificas para a diminuição das disparidades, entre elas: causas violentas, tuberculose, mortalidade materna e infantil, transtornos mentais, cânceres, entre outras. Após sua aprovação, em 2010, a política tornou-se lei, a partir do *Estatuto da Igualdade Racial* ^20^.

## As encruzilhadas do século XX: emancipação e a morte

Considerando os contextos e os fatos já citados, percebe-se o quanto eles se relacionam com a saúde da população negra e a política de morte e/ou emancipatória. Entende-se que, durante o século XX, houve vários mecanismos e ferramentas para o fortalecimento do processo de morte da população negra apoiados pelo Estado, mas ao mesmo tempo houve um processo político emancipatório por parte da população negra e os movimentos negros até os dias atuais. Sendo assim, segue abaixo alguns exemplos de ações e mecanismos que o Estado ainda utiliza para uma política de morte da população negra, além das políticas emancipatórias a partir da luta e resistência dessa população.

Constata-se que o trabalho escravo contemporâneo é uma das políticas de morte empregada pelo Estado, sendo um instrumento econômico adotado como um empreendimento para garantir o lucro na economia globalizada atualmente. Apesar da Lei Áurea, houve persistências nas estratégias de submissão dos trabalhadores. Mesmo com mecanismos diferentes, o trabalho escravo contemporâneo apresenta certas similaridades com o da antiguidade colonial e imperial, pois vigoram o tratamento desumano, a restrição de liberdade e a “coisificação”, consequências diretas de uma abolição incompleta e apoiada pelo Estado. Além disso, é preciso destacar a recente desconstrução de leis que protegem os mais vulneráveis, como a reforma trabalhista, que abriu a possibilidade do aumento da jornada de trabalho, com facilidades de contratação de empreiteiras e restrição das fiscalizações e auditorias ^21^.

Apesar de a ordem escravista continuar ditando as relações sociais, políticas e econômicas, a população negra sempre estabeleceu processos de resistência, organização e combate político de forma coletiva. Dois desses processos, sem dúvidas, são representados pelos quilombos e pelo uso das ervas medicinais até os dias atuais. Tratam-se de elementos que se configuram como política emancipatória, principalmente atravessados pela interseccionalidade, e que nos levam a reconhecer as opressões a partir das articulações e clivagens identitárias, permitindo a não hierarquização do sofrimento. Visto que todo sofrimento está imbricado na estrutura social em que vivemos e, de certa forma, nos orientam, o que faremos politicamente ^22^? É importante reforçar que essa resistência também parte de mulheres quilombolas, seja no período colonial ou na contemporaneidade. “*As mulheres sabem que ter passado é uma necessidade pessoal e coletiva que permite o fortalecimento dos vínculos pessoais e subjetivos, tanto individualmente quanto nos grupos que reivindicam o direito à própria vida*” ^23^ (p. 198).

As práticas das mulheres quilombolas na contemporaneidade mobilizam um território de afetos, configurando-se como uma ação política que exprime manutenção, criação ou redefinição de espaços potencializados nos quilombos. Dessa forma, é importante trazer a perspectiva de um quilombo do presente, reconhecendo as modificações e a força das organizações dessas comunidades ao longo do tempo. As mulheres quilombolas estabeleceram afetos potentes, como o cuidado, a ternura e a capacidade de criar espaços seguros, partir da reconciliação com seus lugares, suas histórias, seus corpos e seus saberes, criando territórios subjetivos e políticos, mobilizando ações em direção a um devir que é feminino, relacional e coletivo ^23^.

Ainda dentro dos processos emancipatórios, apontamos desde já que é necessário o aquilombamento da saúde mental para o reconhecimento de espaços institucionais e comunitários que resgatam e valorizam saberes tradicionais de cuidados, sobretudo reconhecendo os espaços negros como parte da Rede de Atenção Psicossocial (RAPS). É preciso reconhecer o terreiro de Candomblé, a congada, a roda de samba, o tambor de crioula como espaços que também promovem a saúde a partir de dispositivos ancestrais de cuidado ^24^.

Nos equipamentos de saúde mental, não é recorrente a discussão da temática étnico-racial e, quando é discutida, ainda é colocada em segundo plano. Além do mais, existe uma escassez de estudos e pesquisas sobre a loucura e a saúde da população negra, e os equipamentos de saúde nem sempre percebem que a população atendida é majoritariamente negra, distanciando o cuidado das especificidades dessa população, indo pelo caminho oposto ao estabelecido pela PNISPN, que preconiza a equidade a partir da valorização das diferenças e a partir das especificidades dos sujeitos atendidos. Sendo assim, ações de prevenção e promoção de saúde podem transformar os territórios a partir da desnaturalização do preconceito e fortalecer a identidade negra de forma positiva ^24^. Pois se o hospício é colonial, a luta antimanicomial tem que ser antirracista, caso contrário, não será uma luta antimanicomial.

Seguindo esse mesmo caminho de políticas emancipatórias na contemporaneidade, o CNS, em sua *Resolução nº 715/2023* ^25^, reconheceu as manifestações da cultura popular dos povos tradicionais de matriz africana e as Unidades Territoriais Tradicionais de Matriz Africana (terreiros, terreiras, barracões, casas de religião etc.) como equipamentos promotores de saúde e cura complementares do SUS no processo de promoção da saúde. Além disso, esses espaços são considerados primeira porta de entrada para os que mais precisam e locais de cura para o desequilíbrio mental, psíquico, social e alimentar, de modo que é importante respeitar as complexidades inerentes às culturas e povos tradicionais de matriz africana, na busca da preservação desses instrumentos, previstos na política de saúde pública e que contribuem para o combate ao racismo, à violação de direitos, à discriminação religiosa, entre outros ^25^.

Entende-se que ainda existe a continuação de mecanismos a partir da política de morte do Estado. Na contemporaneidade, a população negra continua tendo dificuldades no acesso à saúde por causa do racismo institucional e de suas ferramentas. Segundo o *Relatório Técnico nº 02/2023* ^26^, do Instituto de Estudos para Políticas de Saúde (IEPS), “*o racismo institucional é um dos maiores impeditivos à implementação da PNSIPN*”.

Ainda nesse processo de implementação de uma política de morte, o sistema criou um método de manutenção da hierarquia racial, o colorismo ^27^, que coloca uma indivíduo contra outro, fazendo com que pessoas negras possam se estranhar por causa de suas diferenças. É necessário reconhecer que esse mecanismo está relacionado ao abuso patriarcal do corpo das mulheres negras, considerando os abusos, os estupros e a supervalorização da pele clara. Nesse sentido, o colorismo bloqueia as vias de libertação do povo negro, impedindo um reconhecimento mútuo da diversidade catalizadora das reparações históricas para a transformação da sociedade. Inicialmente, as mulheres negras tiveram uma maternidade forçada, se considerarmos o papel da ama de leite, que fez com que a mulher representasse um acessório reificado para a prole branca, sujeito despido de desejos e humanidade. Após esse processo, ganha uma segunda existência a doméstica, que é privada e dominada pela família branca, perpetuando a reintegração da ex-escrava. Finalmente, temos a existência da “mulata”, passando por precarização do trabalho, prostituição, parceira não assumida ou hipersexualização ^27^.

Durante o século XX, houve processos institucionais, principalmente no caminho da construção de secretarias, institutos, departamentos preventivos, entre outros, inclusive posteriormente à construção do SUS. O Movimento da Reforma Sanitária Brasileira (MRSB) reuniu diferentes atores, como organizações, instituições, sindicatos, movimentos populares, igreja e partidos políticos para a construção do SUS ^28^, porém, mesmo entre as lideranças dos movimentos negros, não há menções em relação à participação direta ou indireta com entidades do MRSB; existe referências fortes de atuação no CNS, na formação de conselheiros de saúde e nas Conferências Nacionais de Saúde ^29^.

Ao contrário da educação, a saúde não teve o fenômeno de intelectuais e professores negros vinculados aos movimentos negros e que produzissem reflexões acadêmicas, uma vez que os cursos de saúde, sobretudo a medicina, são ocupados pelas elites brancas brasileiras - situação em vigor antes da implantação das políticas de ações afirmativas na década de 2000 ^29^.

Por mais que existam políticas de morte, a população negra e os movimentos negros produzem políticas emancipatórias. A partir de ações comunitárias, por meio das entidades de ajuda mútua, associações profissionais de trabalhadores negros (estivadores, marceneiros etc.) e por meio das próprias religiões de matriz africana, que se configuram como uma herança afro-brasileira de organização autônoma da população negra, segue a busca pela resolução das demandas mais imediatas ^29^.

Os saberes emancipatórios da população negra são uma forma de conhecer o mundo. Significa a intervenção social, cultural e política de forma intencional e direcionada dos negros e negras ao longo da história, na vida em sociedade, nos processos de produção e reprodução da existência a partir da criação, recriação, produção e potência ^1^.

Assim, é possível afirmar que houve vários movimentos de resistência acontecendo e sendo silenciados temporariamente em todo o processo, mas que, obstinadamente, estão construindo um mundo possível e com vitalidade, mas a sua dissonância ganha força e gera contradições no sistema ^30^, ou seja, a capacidade emancipatória dos negros e negras parte de saberes identitários, políticos e estético-corpóreos, entrelaçados e interligados, apesar de suas especificidades ^1^.

## Considerações finais

Identifica-se que a relação da saúde da população negra com as políticas de morte e emancipatórias são bem próximas, porém demostram que a consolidação de uma saúde democrática e antirracista para a população negra, ao longo da perspectiva histórica, é algo que caminha a passos lentos.

Foi possível verificar que o Estado sempre produziu políticas e ferramentas de morte para a população negra, de acordo com as perspectivas econômicas e políticas do contexto. Já as políticas emancipatórias são construídas pela luta e resistência da população negra ao longo do tempo, uma dessas construções de luta é a aprovação da PNSIPN, que, posteriormente, se tornou lei, a partir do *Estatuto da Igualdade Racial*.

No decorrer do tempo, as políticas e ferramentas de morte estão se modificando e/ou criando perspectivas a partir dos racismos. Dessa forma, assim como os racismos se atualizam, é necessária uma renovação para a produção de conhecimento e práticas antirracistas na saúde, buscando alternativas democráticas, territorializadas, racialmente críticas e interseccionalizadas. Percebe-se que as políticas emancipatórias são de grande importância para a população negra, pois é uma forma de o Estado diminuir as iniquidades, partindo de uma reivindicação da população negra.

Observa-se que as políticas de morte e emancipatórias não se confundem entre si, mas se articulam e se comunicam de forma dinâmica em prol de um alvo comum (os racismos), seja para potencializá-los, para minimizá-los ou enfrentá-los. É importante compreender as barreiras e zonas de conflitos para que possamos contribuir para ações emancipatórias e resistência democrática, principalmente quando falamos da saúde da população negra.
